# Changes of blood pressure, abdominal visceral fat tissue and gene expressions in fetal programming induced rat model after amlodipine–losartan combination treatment

**DOI:** 10.1186/s40885-016-0046-9

**Published:** 2016-04-05

**Authors:** Ji Hyen Lee, Hyeryon Lee, Sang Mi Lee, Pil Je Kang, Kwan Chang Kim, Young Mi Hong

**Affiliations:** Department of Pediatrics, Ewha Womans University, 911-1, Mokdong, YangCheon-Ku, Seoul, South Korea; Department of Thoracic and Cardiovascular Surgery, Ewha Womans University, Seoul, South Korea

**Keywords:** Fetal programming, Blood pressure, Visceral fat, Gene expression

## Abstract

**Background:**

There are a number of complications that can occur if there is under-nutrition during pregnancy followed by a period of rapid catch-up growth, including a higher chance of adult obesity, insulin resistance and hypertriglyceridemia. The purposes of this study were to investigate the effects of fetal under-nutrition during late pregnancy and lactation on blood pressure, visceral fat tissue, gene expressions and to evaluate changes after amlodipine- losartan combination treatment.

**Methods:**

The rats were divided into three groups: the control (C) group, the food restriction (FR: 50 % food restricted diet) group, and the CX group, which was treated with Cozaar XQ (amlodipine- losartan combination drug) in FR rats from postnatal 4 to 20 weeks. Masson’s trichrome staining was performed in the heart tissues. The amount of abdominal visceral fat tissues was measured. Western blot analysis such as angiotensin converting enzyme (ACE), angiotensin II receptor type IA (ATIA), troponin I (Tn I) and endothelial nitric oxide synthase (eNOS) were performed.

**Results:**

Body weights were significantly higher in the FR group compared with the C group at weeks 8 and 20 and lower in the CX group at week 20. Blood pressure was significantly higher in the FR group compared with the C group at week 20 and lower in the CX group at weeks 12 and 20. The amount of abdominal visceral fat was significantly higher in the FR group compared with the C group at weeks 8, 12 and 20 and significantly lower in the CX group at weeks 16 and 20. Protein expression of ATIA and eNOS were significantly reduced in the CX group at weeks 16 and 20. ACE was significantly reduced in the CX group at week 20 and Tn I was significantly reduced in the CX group at week 16.

**Conclusions:**

When there is fetal under-nutrition during pregnancy, it leads to obesity, high blood pressure, hypertriglyceridemia and several gene changes in offspring. Amlodipine-losartan combination treatment was able to lower obesity, hypertension, hypertriglyceridemia and several gene changes in rats suffering from fetal under-nutrition during pregnancy.

## Background

Fetal programming, due to maternal under-nutrition, is a critical problem, especially in developing countries. It can lead to the development of metabolic syndrome (MS), hypertension, coronary heart disease and diabetes in adulthood [1]. Early prenatal events in the fetus can result in cardiovascular and metabolic pathologies in both in humans and experimental models [2, 3]. Although we know little about the precise mechanisms of fetal programing, some mechanisms have been more extensively studied including glucocorticoid exposure, the role of the kidneys and the renin angiotensin system (RAS) [4, 5]. Other pathophysiological pathways have been investigated, such as the role of the brain and oxidative stress, sympathetic nervous system, and epigenetic changes [4].

Numerous methods have been employed to induce fetal under-nutrition in animal studies such as maternal under-nutrition during pregnancy [6], placental insufficiency [7], or pharmacological manipulations [8].

A reading of the research done in this field mentions a variety of results. We can find evidence in the literature for a link between fetal under-nutrition and an increased risk of MS [3], but other research mentions just components of MS, including hypertension [9], impaired glucose tolerance and insulin resistance [10], dyslipidemia [11, 12], obesity [13] and coronary heart disease [14].

Intrauterine growth retardation may result in a decrease in the expression of genes involved in nephrogenesis [15], as well as a re-directing of nutrients from the kidney to more essential organs such as the brain, heart and adrenal glands in order to increase the survival of the fetus [16]. Sun, et al. reported that offspring exposed to altered maternal nutrition *in utero* showed modulation of gene expression in adult life associated with subsequent hypertension and dyslipidemia [17].

Blockade of the RAS prevents or gets rid of hypertension in fetal programming animal models, so RAS is most-likely very important in the etiology of prenatal programmed hypertension [6, 18]. There have been controversies about the effect of antihypertensive drugs such as losartan and angiotensin converting enzyme (ACE) inhibitors on fetal programming models [19]. There have yet to be any reports on the effects of a fixed-dose combination therapy of amlodipine and losartan (Cozaar XQ) in a fetal programming model. Our paper is the first to study about an effect of amlodipine- losartan combination in a fetal programming model.

The purposes of this study were to investigate the effects of fetal under-nutrition during pregnancy and lactation on abdominal visceral fat, lipid profiles, blood pressure and several genes such as ACE, angiotensin II receptor type IA (ATIA), troponin I (Tn I) and endothelial nitric oxide synthase (eNOS) and evaluate changes after amlodipine- losartan combination treatment.

## Methods

### Animals

Nine to twelve weeks old virgin female Wistar rats (Sankyo Lab Service, Tokyo, Japan) were maintained at 12 h light/12 h darkness cycles with free access to tap water and standard rat chow (laboratory animal diet MF; Oriental Yeast, Tokyo, Japan). Female rats were mated with male Wistar rats and conception was confirmed by the observations of semen plugs on the floor of the mating cage.

Pregnant rats were studied from the 10th day to term gestation and through lactation.

Control pregnant rats were fed *ad libitum* (AdLib) food, whereas the study rats were 50 % food restricted (FR). In this study, only the male offsprings were used. The rats were divided into three groups: the control (C) group (*n* = 20), the food restriction (FR: 50 % food restriction diet (*n* = 40)] group, CX group (*n* = 40), which was treated with Cozaar XQ (amlodipine- losartan combination drug orally) in the FR rats from postnatal 4 to 20 weeks.

Food intake and maternal weights were recorded daily until birth. After birth, body weight was recorded daily. The rats were sacrificed at weeks 4, 8, 12, 16 and 20. The heart and kidney tissues were removed and immediately frozen at −70 °C for protein and gene analysis, post-fixed in 10 % formalin, and processed routinely for paraffin embedding. All protocols were approved by the Institutional Animal Care and Use Committees of the School of Medicine of Ewha Womans University (approval No. 13-0244).

### Organ weights

The rats were weighed and their general appearance was observed daily during the study period. The hearts and kidneys were rapidly removed. The wet weights of the left ventricle (LV) and kidney were measured.

### Estimation of abdominal visceral fat tissues

The amount of abdominal visceral fat (mesenteric, perirenal and pararenal abdominal fat tissues) was estimated at weeks 4, 8, 12, 16 and 20.

### Estimation of systemic blood pressure

The animals were placed in the supine position and instrumented with an arterial pressure line (Physiological Pressure Transducer, MLT 1199; AD Instruments, Oxfordshire, UK). Arterial pressures were estimated at external carotid artery using the input from an ambient-pressure reference (APR-1; Data Sciences) at weeks 4, 8, 12, 16 and 20.

### Morphometric analysis of the heart tissues

The LV including the interventricular septum was weighed after the right and left atria and the right ventricular free wall were dissected. The LV heart tissue was fixed in formaldehyde and embedded as a paraffin section in all three groups.

Masson’s trichrome staining was used in order to observe the degree of fibrosis in the heart tissues. The area with the most and least degree of fibrosis within each group was selected and photographed under 200 HPF light microscopy. The photographs were processed through an image analysis program (analySIS).

### Serum lipid profile

Blood was drawn from all the rats. They fasted for 12 h prior to their blood draw to determine blood levels of the following parameters: blood glucose, total cholesterol (TC), high density lipoprotein-cholesterol (HDL-C), low density lipoprotein-cholesterol (LDL-C), triglyceride (TG) at weeks 4, 8, 12, 16 and 20.

### Westernblot analysis of heart tissues

Whole tissue extracts from rat heart were prepared by homogenization in lysis buffer (Proprep, Intron, Seongnam, Korea). Lysates were normalized and separated on 8–12 % polyacrylamide gels and transferred to nitrocellulose blotting membranes (GE Healthcare: formerly Amersham Bioscience, Munich, Germany). After staining with Ponceau red, membranes blocked with 5 % bovine serum albumin an hour at room temperature followed by incubation with primary antibodies including four genes: ACE, ATIA, Tn I, and eNOS (Santacruz Biotechnology, Santa Cruz, CA, USA) overnight.

Nitrocellulose membranes were then washed in t-TBS. The membranes were incubated with horseradish conjugated secondary antibodies for one hour at room temperature. The bound secondary antibody was detected by ECL™ Western blotting detection reagents (GE Healthcare: formerly Amersham Bioscience, Buckinghamshire, UK).

### Statistical analysis

Results are expressed as the mean ± standard deviation. Data were analyzed using SPSS (SPSS v22.0, Chicago, IL, USA) for windows. The Mann-Whitney test was used to test for significant differences between the C and FR group. The Kruskal-Wallis test was used to test for significant differences between FT group and CX group.

## Results

### Body weights

There was no significant change in body weight at week 4 among the groups.

Body weight was significantly increased at week 8 in the FR group compared with the C group (C vs. FR; 302.50 ± 16.83 g vs. 369.25 ± 28.15 g, *p* < 0.05), and at week 20 (C vs. FR; 553.50 ± 13.52 g vs. 582.10 ± 19.70 g, *p* <0.05). Body weight was significantly decreased in the CX group compared with the FR group at week 20 (FR vs. CX; 582.10 ± 19.70 g vs. 552.30 ± 1.09 g, *p* <0.05) (Table 1).

**Table 1:** Changes of body weight in fetal programming model after amlodipine-losartan combination treatment

Week	C (g)	FR (g)	CX (g)
4	90.75 ± 8.33	85.66 ± 6.79	97.67 ± 21.26
8	302.50 ± 16.83	369.25 ± 28.15*	333.13 ± 27.54
12	395.125 ± 26.575	408.67 ± 24.95	400.875 ± 11.89
16	512.38 ± 20.90	541.50 ± 35.64	507.50 ± 15.21
20	553.50 ± 13.52	582.10 ± 19.70*	552.30 ± 1.09†

*C* control, *FR* food restriction, *CX* amlodipine- losartan combination

*C group vs FR group, *p* < 0.05, † FR group vs CX group, *p* < 0.05

### Abdominal fat tissues

**Fig. 1:**
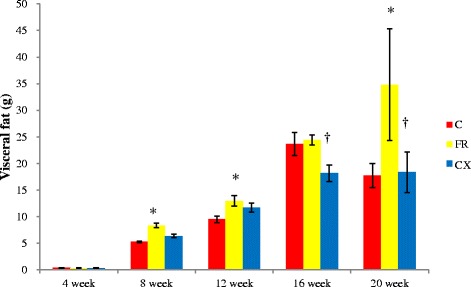
Abdominal fat tissues in fetal programming model after amlodipine–losartan combination treatment. Abdominal fat tissues were significantly reduced in the CX group compared with the FR group at weeks 16 and 20. C, control; FR, food restriction; CX, Cozaar XQ. *p <0.05 C group vs FR group, † *p* <0.05 FR group vs CX group

Abdominal fat tissues were significantly increased in the FR group compared with the C group at week 8 (C vs. FR; 5.26 ± 0.14 g vs. 8.38 ± 0.45 g, *p* < 0.05), 12 (C vs. FR; 9.54 ± 0.59 g vs. 12.96 ± 1.01 g, *p* < 0.05), and 20 (C vs. FR; 17.77 ± 2.23 g vs. 34.83 ± 10.47 g, *p* < 0.05) (Fig. 1).

Abdominal fat tissues were significantly reduced in the CX group compared with the FR group at weeks 16 (FR vs. CX; 24.44 ± 0.93 g vs. 18.20 ± 1.55 g, *p* < 0.05) and 20 (FR vs. CX; 34.83 ± 10.47 g vs.18.36 ± 3.79 g, *p* < 0.05), (Fig. 1).

### Blood pressure

Blood pressure was significantly increased in the FR group compared with the C group at week 20 (119.80 ± 26.22 mmHg vs. 141.75 ± 19.70 mmHg) (*p* < 0.05, Table 2).

**Table 2:** Changes of blood pressure in fetal programming model after amlodipine-losartan combination treatment

Week	C (mmHg)	FR (mmHg)	CX (mmHg)
4	86.00 ± 12.73	82.50 ± 10.61	84.25 ± 6.24
8	100.00 ± 16.57	113.25 ± 5.32	101.75 ± 11.76
12	103.25 ± 6.99	122.00 ± 9.9	97.00 ± 1.73†
16	109.50 ± 3.32	103.67 ± 13.58	110.25 ± 10.24
20	119.80 ± 26.22	141.75 ± 19.70*	121.00 ± 00.00†

*C* control, *FR* food restriction, *CX* amlodipine-losartan combination

*C group vs FR group, *p* <0.05, † FR group vs CX group, *p* <0.05

Blood pressure was significantly decreased in the CX group compared with the FR group at week 12 (FR vs. CX; 122.00 ± 9.9 mmHg vs.97.00 ± 1.73 mmHg and 20 (FR vs. CX; 141.75 ± 19.70 mmHg vs. 121.00 mmHg), *p* <0.05, Table 2).

### LV + S/RV ratio

There was no significant difference among the groups. Data was not shown.

### Pathologic finding in heart tissues

**Fig. 2:**
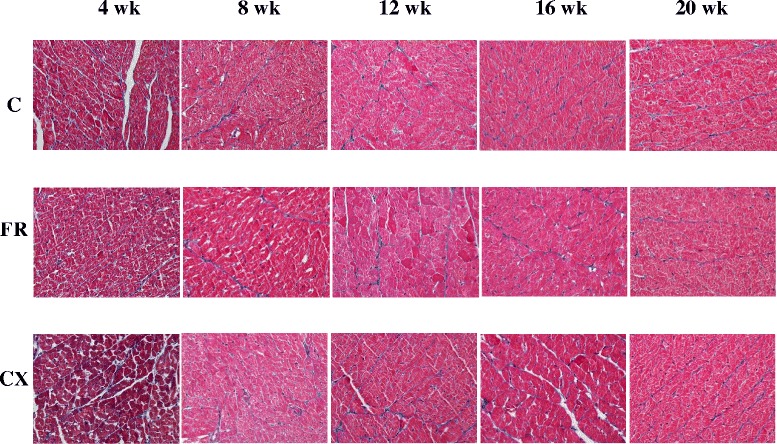
Pathologic finding in the heart tissues by Masson’s trichrome staining. There was not significantly different between groups. C, control; FR, food restriction; CX, Cozaar XQ

Under light microscopy, collagen was observed to penetrate between the LV myocardiocytes, staining blue as noted in Fig. 2. The degree of collagen was not significantly different in each group.

### Westernblot analysis

**Fig. 3:**
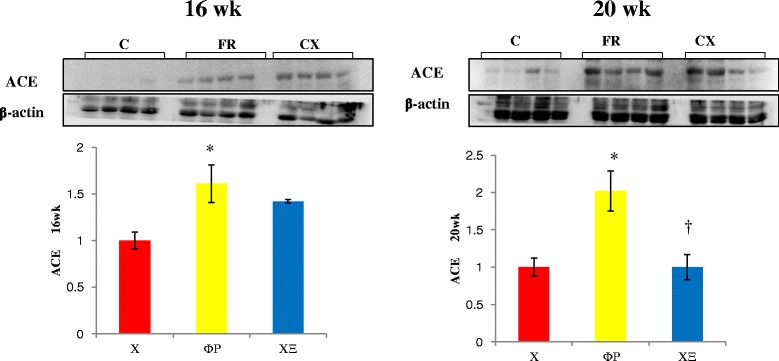
ACE protein in the heart tissues by westernblot analysis. Protein expressions of ACE were significantly decreased in the CX group compared with the FR group at week 20. ACE, angiotensin converting enzyme; C, control; FR, food restriction; CX, Cozaar XQ. *p <0.05 C group vs FR group, † *p* <0.05 FR group vs CX group

**Fig. 4:**
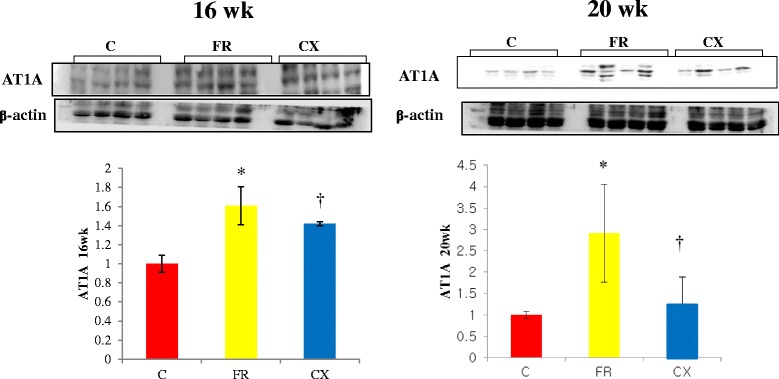
AT1A protein in the heart sissues by westernblot analysis. Protein expressions of AT1A were significantly decreased in the CX group compared with the FR group at weeks 16 and 20. AT IA; angiotensin receptor type IA; C, control; FR, food restriction; CX, Cozaar XQ. *p <0.05 C group vs FR group, † *p* <0.05 FR group vs CX group

**Fig. 5:**
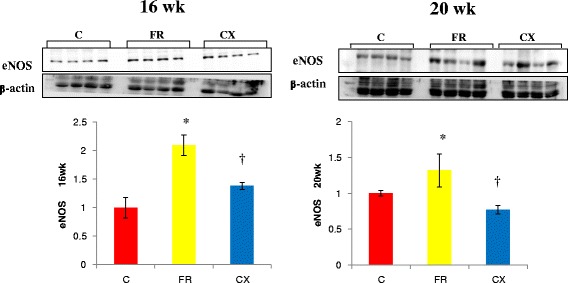
eNOS protein in the heart tissues by westernblot analysis. Protein expressions of eNOS were significantly decreased in the CX group compared with the FR group at weeks 16 and 20. eNOS, endothelial nitric oxide synthase; C, control; FR, food restriction; CX, Cozaar XQ. *p <0.05 C group vs FR group, † *p* <0.05 FR group vs CX group

**Fig. 6:**
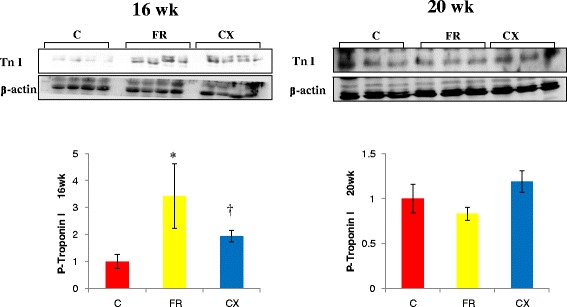
Troponin I protein in the heart tissues by westernblot analysis. Protein expressions of Tn I were significantly decreased in the CX group compared with the FR group at week 16. Tn; troponin; C, control; FR, food restriction; CX, Cozaar XQ. *p <0.05 C group vs FR group, † *p* <0.05 FR group vs CX group

Protein expressions of ACE (Fig. 3), AT1A (Fig. 4) and eNOS (Fig. 5) were significantly increased in the FR group compared with the C group at weeks 16 and 20. Protein expressions of Tn I was significantly increased in the FR group compared with the C group at week 16 (Fig. 6).

Protein expressions of ACE were significantly decreased in the CX group compared with the FR group at week 20 (Fig. 3). Protein expressions of AT1A (Fig. 4) and eNOS (Fig. 5) were significantly decreased in the CX group compared with the FR group at weeks 16 and 20. Protein expressions of Tn I were significantly decreased in the CX group compared with the FR group at week 16 (Fig. 6).

### Serum lipid profile

TG was significantly higher at weeks 8, 12 and 20 in the FR group compared with the C group and decreased in the CX group compared with FR group at week 20 (76.80 ± 13.03 mg/dL vs. 42.75 ± 10.34 mg/dL, *p* < 0.05).

TC, HDL-C and LDL-C were not significantly different between groups (Table 3).

**Table 3:** Changes of serum lipid profile in fetal programming model after amlodipine-losartan combination treatment

Week		Blood sugar (mg/dL)	TG (mg/dL)	TC (mg/dL)	HDL-C (mg/dL)	LDL-C (mg/dL)
4	C	288.50 ± 142.13	107.50 ± 62.98	84.33 ± 3.06	65.33 ± 11.93	18.00 ± 6.08
	FR	237.00 ± 108.43	142.00 ± 47.76	82.67 ± 2.52	66.67 ± 0.87	16.00 ± 4.58
	CX	205.00 ± 0.00	83.00 ± 2.83	76.50 ± 2.12	65.50 ± 0.72	18.00 ± 0.00
8	C	202.00 ± 14.72	45.75 ± 14.24	43.00 ± 6.78	40.50 ± 6.24	4.50 ± 1.00
	FR	207.50 ± 58.34	145.50 ± 26.80*	62.75 ± 8.34	56.75 ± 0.85	7.50 ± 1.73
	CX	263.00 ± 25.87	105.50 ± 28.63	62.75 ± 7.04	57.50 ± 5.45	9.25 ± 1.89
12	C	127.75 ± 10.21	20.25 ± 10.31	42.25 ± 3.40	42.25 ± 2.99	5.75 ± 1.26
	FR	101.00 ± 18.17	38.25 ± 7.50*	41.50 ± 7.59	42.00 ± 6.68	5.00 ± 2.16
	CX	165.00 ± 94.69	30.00 ± 8.54	37.25 ± 22.51	37.00 ± 14.72	4.25 ± 3.86
16	C	169.33 ± 3.33	62.50 ± 14.50	60.50 ± 12.82	52.00 ± 10.39	4.67 ± 0.47
	FR	189.50 ± 11.32	81.50 ± 0.32	54.25 ± 6.54	48.50 ± 12.84	5.00 ± 2.74
	CX	191.67 ± 39.14	86.25 ± 32.93	54.50 ± 7.09	445.75 ± 4.44	5.00 ± 0.82
20	C	141.17 ± 36.99	33.20 ± 13.31	48.50 ± 10.88	45.17 ± 9.70	5.33 ± 2.42
	FR	195.00 ± 22.58	76.80 ± 13.03*	63.71 ± 17.74	56.71 ± 16.54	6.17 ± 3.29
	CX	181.00 ± 19.61	42.75 ± 10.34†	68.00 ± 24.99	63.40 ± 2.48	8.20 ± 3.49

*C* control, *FR* food restriction, *CX* amlodipine losartan-combination, *TG* triglyceride, *TC* total cholesterol, *HDL-C* high density lipoprotein cholesterol, *LDL-C* low density lipoprotein cholesterol

*C group vs FR group, *p* <0.05, † FR group vs CX group, *p* <0.05

## Discussion

Our research confirmed that fetal under-nutrition during pregnancy resulted in obesity, high blood pressure, hypertriglyceridemia and several gene changes in the offspring.

Body weight was significantly increased at weeks 8 and 20 in the FR group. BP was significantly increased in the FR group at week 20. Abdominal visceral fat was significantly larger at weeks 8, 12 and 20 in the FR group. Protein expressions of ACE, AT1A, eNOS and Tn I were significantly increased in the FR group. TG was significantly higher in the FR group at weeks 8, 12 and 20.

Effects of amlodipine-losartan combination on fetal programming model were as follows.

Body weight was significantly reduced in the CX group compared with the FR group at week 20. Abdominal fat tissues were significantly reduced in the CX group at weeks 8, 12, 16 and 20. BP was significantly decreased in the CX group at weeks 12 and 20. Protein expressions of ACE, AT1A, eNOS and Tn I were significantly decreased in the FR group. TG was significantly decreased in the CX group at week 20.

In our research, amlodipine-losartan combination treatment in fetal programming model reduced obesity, hypertension, hypertriglyceridemia and gene changes.

There has been some controversy related to the results of fetal programing depending on the investigator. We’ve noticed differences in the literature for the timing of the appearance of elevated BP, as well as its persistence. Most of the research notes elevated BP in animal models between 9 and 12 weeks of age using low protein diet (LPD), reduced uterine perfusion [20] or globally restricted diet [21].

But, the occurrence of the increased BP was different in some research. In the offspring of dams subjected to a 50 % caloric reduction, BP is significantly increased at 4 weeks of age [20]. Vehaskari, et al. reported that after 12 weeks of age, BP either remained unchanged or continued to elevate up to 10 months of age [21]. In a Jansson study, BP appears to diminish as the subject gets older [22]. In our research, BP was significantly increased in the FR group compared with the C group at week 20.

Wintour, et al. noted that there was a difference in BP in fetal programming models, persisting in subjects for up to 7 years of age [8].

The mechanisms of hypertension in fetal programming models are multi-factorial and complex. They include changes of the RAS [23]. Sahajpal, et al. have reported that renal AT1 receptor expressions increased by 24 % in offspring of LPD dams [24]. This suggests that activation of the RAS is one of the most important determinants in hypertension induced by maternal calorie restriction. This finding was similar with our data.

Also, changes of lipid metabolism on fetal programming are different in some investigations [12, 19]. Intrauterine under-nutrition has been linked with an elevated risk of dyslipidemia and insulin resistance, a decrease in insulin content [12] and hyperglycemia in female offspring [25] .

Lucas, et al. noted that dams who were provided with a LPD in both pregnancy and lactation had offspring that at a mean of 6 months had a significant reduction in plasma concentrations of TC, HDL-C and TG compared with the control group [26]. However, Desai, et al. showed that 6-month-old offspring given 50 % food restriction during pregnancy and the lactation period lead to higher TG and normal TC concentrations than the control group [27]. In our research, serum TG was significantly increased in our fetal programming model at weeks 8, 12 and 20.

Mechanism involved in fetal programming may depend on the duration and timing of nutritional insult [3]. When maternal under-nutrition creates *in utero* stress, the developing fetus may reprogram the genome to overcome the new, more dangerous environment. This may favor immediate survival but it generally results in a predisposition to metabolic disease in adult life. The development of subsequent chronic disease in adulthood may depend on the amount of difference between pre and postnatal environment [28].

In another study, increased methylation of retinoid X receptor and eNOS were linked to larger adiposity in later childhood which suggests that epigenetics plays a role in fetal programming [29].

Several animal studies have suggested that fetal nutrition acts as epigenetic stimuli to bring about changes in the regulation of gene expression [3]. In our research and some of other research, the epigenetic adaptation persisted from 2 weeks to 4 months of age, which lead to a prediabetic state in the rats [3]. In our research, several gene expressions such as ACE, ATIA, eNOS and Tn I were significantly increased in the FR group.

Glucocorticoid steroids may play a key role in the fetal origins of hypertension, because the increased BP in offspring of LPD dams depends not only on an intact adrenal gland in postnatal life [23], but also on maternal glucocorticoid synthesis during pregnancy [30]. Glucocorticoid steroids may up-regulate the actions of the RAS at the level of angiotensinogen synthesis, ACE, and more importantly, the AT 1 receptor [23, 31].

The effects of antihypertensive drugs on fetal programming model are different according to investigators. Sherman, et al. administrated the ACE inhibitor captopril, for a period of 18 days, beginning at 23 weeks after birth. This gets rid of the hypertension of LPD offspring for some 7–8 weeks after the cessation of treatment [32]. Sherman, et al. have also previously demonstrated that the specific AT1 antagonist losartan at 2–4 weeks of age prevents an increase in BP in offspring of LPD fed dams during pregnancy [32]. We induced the fetal programing model using food restriction during pregnancy and lactation period. We planned losartan treatment at the pilot study from postanatal 4 week to 20 week. There was no response to reduce blood pressure and gene change after losartan treatment postnatally. Therefore, we decided on a postnatally amlodipine-losartan combination in fetal programming models. The amlodipine- losartan combination treatment reduced obesity, hypertension, hypertriglyceridemia and gene changes such as ACE, AT IA, eNOS and Tn I in our study.

In our previous study, we studied changes of gene expressions in a spontaneously hypertensive rat model after losartan treatment. Systolic blood pressure was significantly decreased in the losartan group compared with the hypertension group in weeks 3 and 5. ACE and ATIA proteins in the losartan group were lower than hypertension group in week 5 [33].

Losartan treatment has been reported to significantly reduce body weight in low-protein-exposed rats, but not in animals fed a control maternal diet (18 % casein).

At 12 weeks of age, low-protein-exposed rats treated with losartan at a young age remained normotensive and had body weights similar to those of untreated rats exposed to 9 % casein diets [31]. In our research, amlodipine- losartan combination treatment from week 4 until week 20 decreased BP, body weight and abdominal fat tissues.

Ceravolo, et al. wrote that at 14 weeks of age, losartan (10 mg/kg, for 15 days) or enalapril treatments stabilized the BP levels and reduced the response to Ang II [19].

Sherman and Langley- Evans [32] found that hypertension does not develop at eight weeks after captopril treatment in a LPD exposed fetal model at postnatal weeks 2–4. This length of treatment could have been sufficient to inhibit the intrarenal angiotensin system, but it was too late in rat renal development to be able to affect nephrogenesis.

Local RAS in the heart, brain and kidney may play a critical role in determining BP.

There are some controversies about the result of ACE activities. Ceravolo, et al. reported no alterations on plasma or tissue ACE activity in intrauterine undernourished rats [19]. However, Langley-Evans SC, et al. have found increased plasma ACE activity in rats exposed to a LPD *in utero* [34]. These discrepancies observed might be due to differences in the feeding protocol used.

In our study, ACE protein expressions were significantly increased in the FR group and reduced after amlodipine- losartan combination treatment.

Amlodipine-losartan combination have become an exciting therapy for patients with hypertension who have cardiovascular co-morbidities [35]. The advantages of combining amlodipine and losartan include a possible lower incidence rate of side effects such as edema, and increased compliance because of its ease of dosing and its improved tolerability, which may also contribute to greater BP-lowering effects [35] .

The main purpose of fixed-dose combination antihypertensive medications is to significantly improve drug intake, thus improving patient adherence, and, ultimately, to maximize the clinical efficacy and minimize the adverse effects found in monotherapy.

Experimental research suggest that it is the timing of the prenatal insult that is critical to the development of hypertension [6]. Animal studies show that when the prenatal insult occurs during the nephrogenic period, significant increases in mean arterial pressure are noted [6, 36].

Both clinical and experimental evidence suggest that sex may have an important impact on the development of cardiovascular disease that may reflect the regulation of the RAS by gonadal hormones including testosterone and estrogen [2]. Therefore, we performed research in only male offspring to exclude the hormone effect on fetal programming.

Studies into the mechanisms involved in the alterations of endothelium-dependent responses is critical for understanding the pathways by which intrauterine under-nutrition leads to endothelium dysfunction and therefore to the development of hypertension and other cardiovascular diseases. Franco, et al. reported that reduced activity of NOS has been described in intrauterine undernourished rats [37]. This result is different from our data. In our study, protein expressions of eNOS were significantly increased in the FR group and significantly decreased after amlodipine-losartan combination treatment.

## Conclusions

In conclusion, we established fetal programming model. Fetal under-nutrition only during late pregnancy resulted in obesity, high blood pressure, hypertriglyceridemia and several gene changes in offspring. Amlodipine-losartan combination treatment reduced obesity, hypertension, hypertriglyceridemia and the changes in genes.

## Abbreviations

C: Control
CX: Cozaar XQ
FR: Food restriction
HDL-C: High density lipoprotein cholesterol
LDL-C: Low density lipoprotein cholesterol
LPD: Low protein diet
TC: Total cholesterol
TG: Triglyceride
